# Identifying the hub genes for Duchenne muscular dystrophy and Becker muscular dystrophy by weighted correlation network analysis

**DOI:** 10.1186/s12863-021-01014-w

**Published:** 2021-12-18

**Authors:** Junjie Wang, Qin Fan, Tengbo Yu, Yingze Zhang

**Affiliations:** 1 Qingdao University, No.308, Ningxia Road, Qingdao, Shandong Province 266000 China; 2 Orthopaedic Center, The Affiliated Hospital of Qingdao University, No. 16, Jiangsu Road, Qingdao, Shandong Province 266000 China; 3 Department of Orthopaedic Surgery, Third Hospital of Hebei Medical University, No. 139, Ziqiang Road, Shijiazhuang, Hebei Province 050000 China

**Keywords:** Duchenne muscular dystrophy, Becker muscular dystrophy, Gene expression omnibus, Weighted correlation network analysis

## Abstract

**Background:**

The goal of this study is to identify the hub genes for Duchenne muscular dystrophy (DMD) and Becker muscular dystrophy (BMD) via weighted correlation network analysis (WGCNA).

**Methods:**

The gene expression profile of vastus lateralis biopsy samples obtained in 17 patients with DMD, 11 patients with BMD and 6 healthy individuals was downloaded from the Gene Expression Omnibus (GEO) database (GSE109178). After obtaining different expressed genes (DEGs) via GEO2R, WGCNA was conducted using R package, modules and genes that highly associated with DMD, BMD, and their age or pathology were screened. Gene Ontology (GO) and Kyoto Encyclopaedia of Genes and Genomes (KEGG) enrichment analysis and protein–protein interaction (PPI) network analysis were also conducted. Hub genes and highly correlated clustered genes were identified using Search Tool for the Retrieval of Interacting Genes (STRING) and Cystoscape software.

**Results:**

One thousand four hundred seventy DEGs were identified between DMD and control, with 1281 upregulated and 189 downregulated DEGs. Four hundred and twenty DEGs were found between BMD and control, with 157 upregulated and 263 upregulated DEGs. Fourteen modules with different colors were identified for DMD vs control, and 7 modules with different colors were identified for BMD vs control. Ten hub genes were summarized for DMD and BMD respectively, 5 hub genes were summarized for BMD age, 5 and 3 highly correlated clustered genes were summarized for DMD age and BMD pathology, respectively. In addition, 20 GO enrichments were found to be involved in DMD, 3 GO enrichments were found to be involved in BMD, 3 GO enrichments were found to be involved in BMD age.

**Conclusion:**

In DMD, several hub genes were identified: C3AR1, TLR7, IRF8, FYB and CD33(immune and inflammation associated genes), TYROBP, PLEK, AIF1(actin reorganization associated genes), LAPTM5 and NT5E(cell death and arterial calcification associated genes, respectively). In BMD, a number of hub genes were identified: LOX, ELN, PLEK, IKZF1, CTSK, THBS2, ADAMTS2, COL5A1(extracellular matrix associated genes), BCL2L1 and CDK2(cell cycle associated genes).

## Background

Duchenne muscular dystrophy (DMD) and Becker muscular dystrophy (BMD) are X-linked recessive diseases, the major genetic alterations are mutations in dystrophin gene [1]. Dystrophin is a part of the dystrophin-glycoprotein complex (DGC), which provides structural stability at the sarcolemma during muscle contraction by linking the internal cell cytoskeleton and external extracellular matrix [2]. Mutations in dystrophin gene can lead to reduction, abnormal or absence of DGC, as a result, degeneration in neuromuscular function occurs [3]. The main symptom of DMD is progressive muscle weakness. DMD patients usually present symptom by age 3 to 5 years, and they successively lose lower and upper limbs function before their adulthood, the most common causes of death for DMD include respiratory and cardiac failure by their 20 to 30 years. It is estimated that nearly half number of patients fail to live to their 20 years old [4]. Compared with DMD, the symptoms of BMD are similar but relatively milder [5]. In addition, the onset, progression, presentation and severity of BMD seem to be more heterogeneous among patients. For example, time point for loss of ambulation ranging from 16 years old to 70 years old [6]. According to worldwide history of newborn screening, the incidence for DMD ranges from 1 in 4589 to 6291 livebirths, and most of them are males, rating as the most common form of muscular dystrophy in children [7]. While according to a meta-analysis, the incidence of BMD is about 2.21 in 100,000 livebirths and males are also the most affected [8].

Currently, the major management for DMD and BMD remains symptomatic treatment such as corticosteroids therapy, wheelchair, ventilation, cough assists and treatment of cardiomyopathy [9]. Although these care can extend life expectancy to some extent [10], DMD and BMD patients still need more effective therapy to treat diseases in order to improve their life quality. Since DMD and BMD are genetic disorders, gene-targeted therapy seems to be a feasible method. However, it is reported that genetic therapy is not usually helpful for a patient who has already lost a substantial part of his muscle tissue and function [11]. Therefore, it is necessary to explore hub genes in order to deeply understand genetic etiology and provide new insights into the early diagnosis and treatment that can be targeted in the pharmaceutical strategy. To the best of our knowledge, although a recent article has identified hub genes for DMD and BMD via weighted correlation network analysis (WGCNA) [12], it is still necessary to identify hub genes for DMD and BMD via WGCNA using differentially expressed genes (DEGs).

In this article, we aim to explore the hub genes for DMD and BMD via WGCNA using DEGs.

## Materials and methods

### Data collection

The gene expression profiles of patients with DMD and BMD, and healthy control were downloaded from the Gene Expression Omnibus (GEO) database. The GSE109178 microarray dataset was used for bioinformatic analysis. GSE109178 (GPL570, Affymetrix Human Genome U133 Plus 2.0 Array) used vastus lateralis biopsy samples obtained from 17 patients with DMD, 11 patients with BMD and 6 healthy individuals.

### Identifying DEGs

GEO2R is an online tool for identifying differentially expressed molecules across various experimental conditions, and it was utilized to identify DEGs between DMD vs control and BMD vs control DEGs were defined from analysis of the microarray data with adjusted *P* value < 0.05 and |log2 fold change (FC)| > 1.5 as cutoffs. The normalization of datasets and limma precision weight analysis were also conducted with GEO2R.

### WGCNA

After obtaining DEGs via GEO2R, WGCNA was conducted using an R package. The adjacency matrix was converted into a topological overlap matrix (TOM). A soft-thresholding power was set, and DEGs were divided into different modules. Modules and clustered genes that were highly associated with DMD, BMD or their age and pathology (such as mild, moderate or severe symptom) were screened (|correlativity| > 0.5).

### Gene ontology (GO) and Kyoto encyclopedia of genes and genomes (KEGG) pathway enrichment analyses

GO is a major bioinformatics tool for annotating genes and analysing their biological processes. KEGG is a database resource for understanding the high-level functions and biological systems of large-scale molecular data generated by high-throughput experimental technologies. To deeply explore the biological functions of highly correlated clustered DEGs between DMD vs control and BMD vs control, WebGestalt (http://www.webgestalt.org/) version 2019, a functional enrichment analysis web tool, was used to conduct GO and KEGG pathway enrichment analyses. A false discovery rate (FDR) ≤0.05 was considered statistically significant.

### Protein–protein interaction (PPI) network construction and hub genes identification

Search Tool for the Retrieval of Interacting Genes (STRING; http://string-db.org) (version 11.0), a web-based tool that analyses the functional interactions among proteins, was used to build a PPI network of the highly correlated clustered DEGs. Cytoscape is an open source software platform for visualizing complex networks and combining them with any type of attribute data. The information in STRING was imported into Cytoscape (version 3.7.1), and the PPI network of highly correlated clustered DEGs was established. The top 10 hub genes were identified according to 12 algorithms.

## Results

### Normalization of dataset

**Fig. 1:**
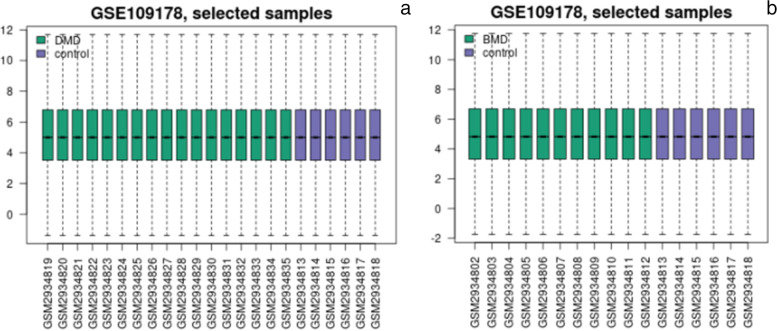
Results of normalization of dataset. **a** DMD vs control; **b** BMD vs control

Figure 1a and b shows the results of the normalization of the dataset, which indicate a relatively high consistency between groups.

### Identification of DEGs

**Fig. 2:**
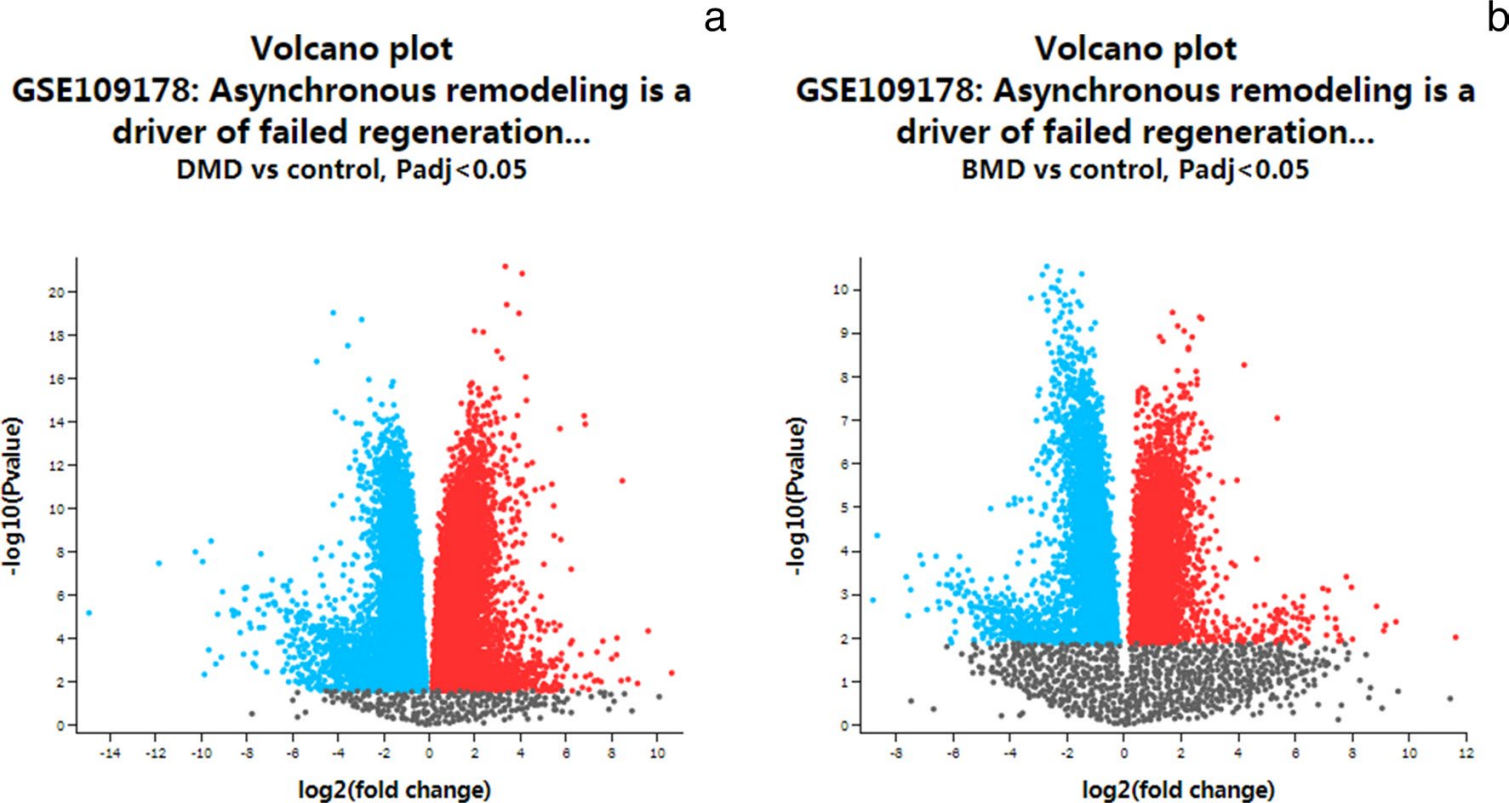
Volcano plots of all DEGs. **a** DMD vs control; **b** BMD vs control

After deleting pseudogene, 1470 DEGs between DMD and control were identified, with 1281 upregulated genes and 189 downregulated genes for DMD. Four hundred and twenty DEGs between BMD and control were found, with 157 upregulated genes and 263 downregulated genes for BMD (Fig. 2a and b).

### WGCNA

**Fig. 3:**
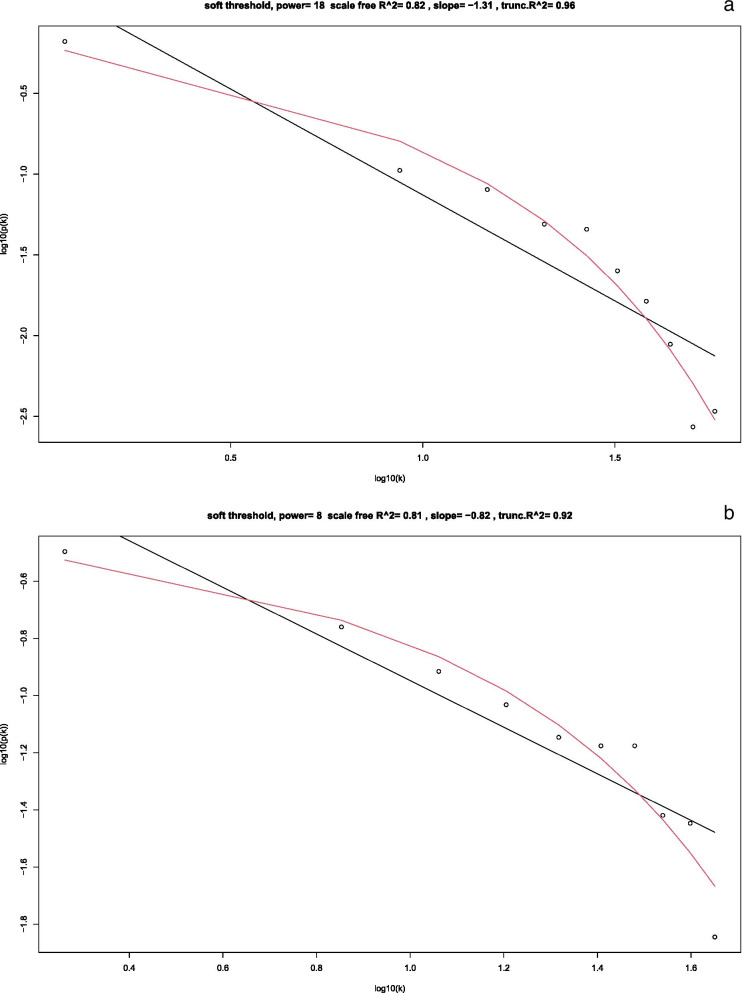
Determination of the soft-threshold powers (β). **a** DMD vs control; **b** BMD vs control

**Fig. 4:**
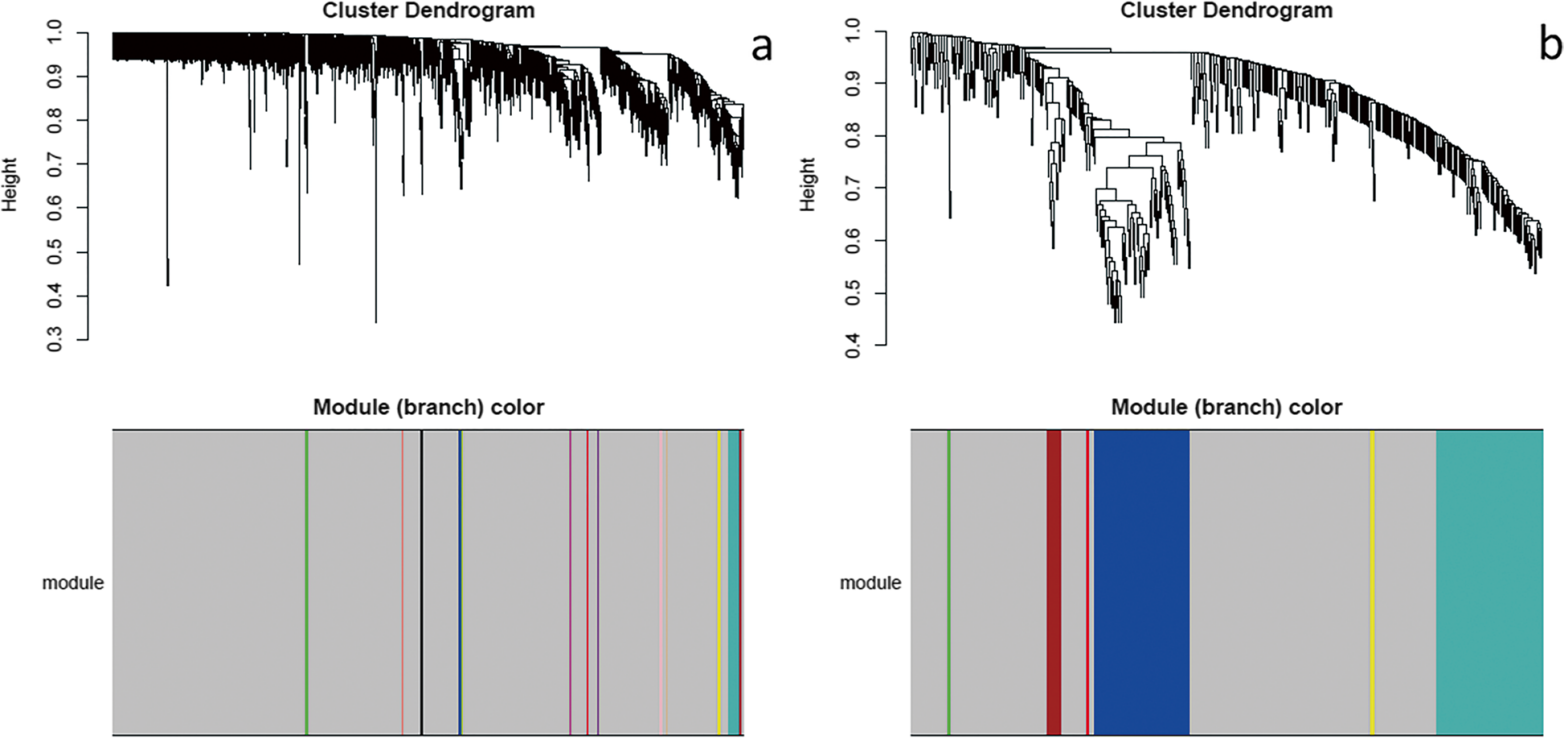
The clustering dendrogram of DEGs, non-clustering DEGs were shown in grey. **a** DMD vs control; **b** BMD vs control

**Fig. 5:**
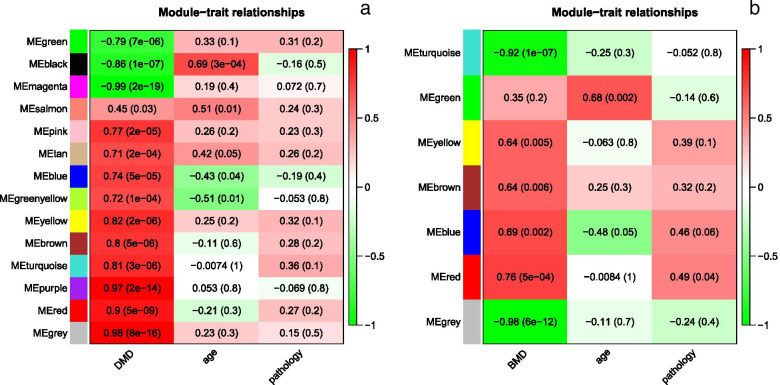
Module-trait relationships. **a** DMD vs control; **b** BMD vs control

The DEGs were then assessed with WGCNA. Figure 3a and b shows that the soft-thresholding power was determined to be β = 18, at which point the curve first achieved Rˆ2 = 0.82 for DMD vs control, andβ = 8 at which point the curve first achieved R^2 = 0.81 for BMD vs control. Subsequently, a TOM-based dissimilarity measure was applied, 14 modules with different colours were identified for DMD vs control, and 7 modules with different colours were identified for BMD vs control, as presented in the dendrogram plots (Fig. 4a and b). In addition, correlation plots between the module colours or genes and clinical traits was constructed (Fig. 5a and b).

### GO and KEGG pathway enrichment analyses

**Fig. 6:**
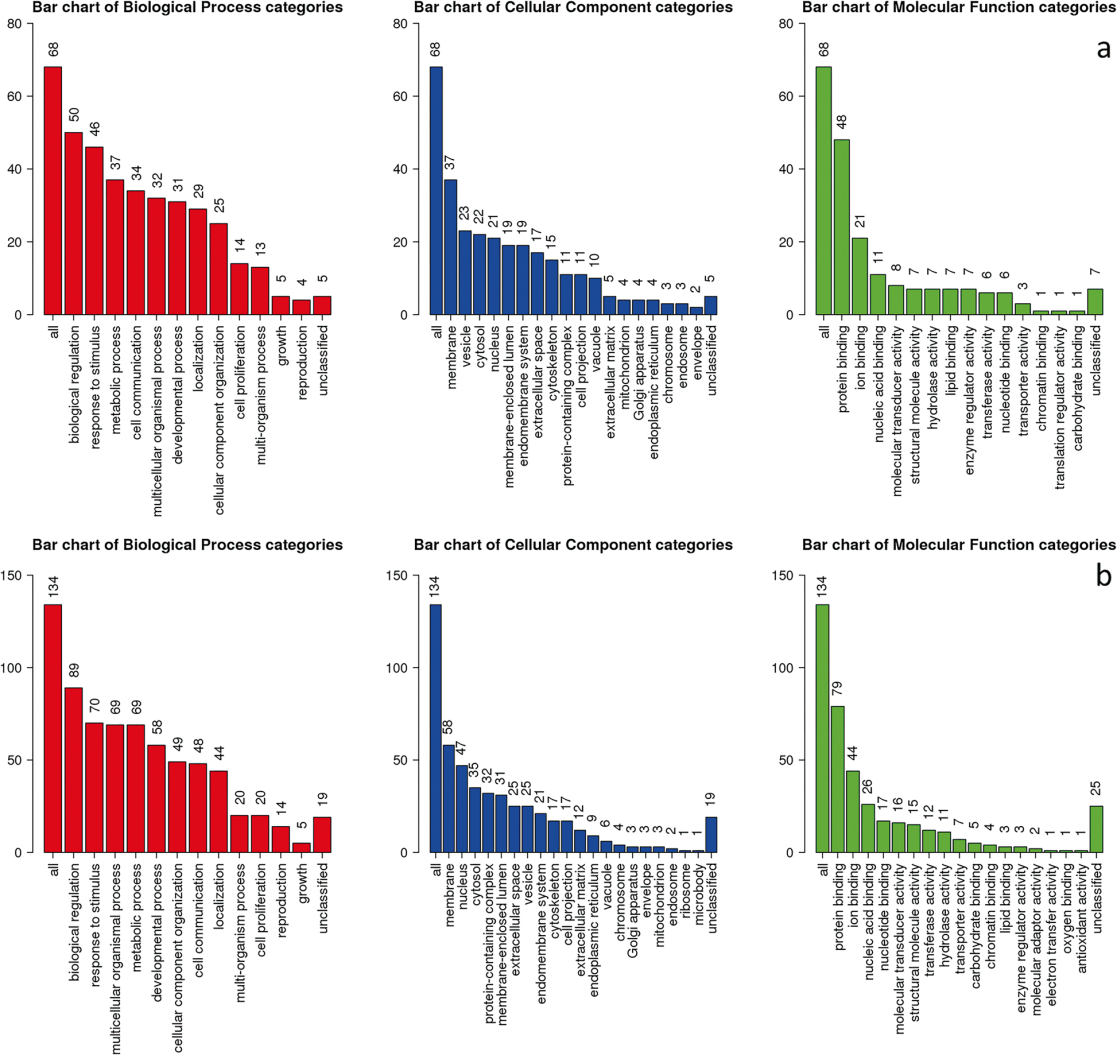
GO enrichment analysis of the highly correlated clustered DEGs. **a** DMD vs control; **b** BMD vs control

The results of GO enrichment analysis for the highly correlated clustered DEGs are shown in Fig. 6a and b. The specific enrichment results showed that for DMD, genes were enriched in immune response, myeloid leukocyte activation, regulated exocytosis, cell activation, neutrophil degranulation, neutrophil activation involved in immune response, defense response, neutrophil activation, neutrophil mediated immunity, and granulocyte activation in the biological process (BP) category, secretory vesicle, secretory granule, lysosome, lytic vacuole, vacuolar part, cytoplasmic vesicle part, vacuole, ruffle, vacuolar lumen, and whole membrane in cellular component (CC) category. For BMD, genes were enriched in extracellular matrix and collagen-containing extracellular matrix in CC category, extracellular matrix structural constituent in molecular function (MF) category. For BMD age, genes were enriched in extracellular matrix and collagen-containing extracellular matrix in CC, extracellular matrix structural constituent in MF category. FDR was more than 0.05 in all KEGG pathway enrichment items for DMD, BMD, age and pathology.

### PPI network and hub genes

The PPI network of the highly correlated clustered DEGs was constructed using STRING and then imported into Cytoscape. Using the 12 algorithms in the CytoHubba plugin, hub genes or highly correlated genes for DMD, BMD, age and pathology were summarized (Table 1). The GO and KEGG pathway enrichment analysis that hub genes were enriched were summarized in Table 2.

**Table 1:** Hub genes or highly correlated genes for clinical traits

Clinical traits	Gene symbol	Module	Positive or negative	Remarks	Clinical traits	Gene symbol	Module	Positive or negative	Remarks
DMD	TYROBP	Yellow	Positive	Hub gene	BMD	LOX	Blue	Positive	Hub gene
	C3AR1	Turquoise	Positive	Hub gene		ELN	Blue	Positive	Hub gene
	PLEK	Yellow	Positive	Hub gene		PLEK	Blue	Positive	Hub gene
	TLR7	Turquoise	Positive	Hub gene		BCL2L1	Turquoise	Negative	Hub gene
	LAPTM5	Brown	Positive	Hub gene		IKZF1	Blue	Positive	Hub gene
	AIF1	Yellow	Positive	Hub gene		CTSK	Blue	Positive	Hub gene
	IRF8	Turquoise	Positive	Hub gene		THBS2	Blue	Positive	Hub gene
	FYB	Turquoise	Positive	Hub gene		ADAMTS2	Blue	Positive	Hub gene
	CD33	Turquoise	Positive	Hub gene		CDK2	Turquoise	Negative	Hub gene
	NT5E	Tan	Positive	Hub gene		COL5A1	Blue	Positive	Hub gene
DMD age	KRT31	Black	Positive	Correlated genes	BMD age	COL14A1	Blue	Negative	Hub gene
	KRT33A	Black	Positive	Correlated genes		ECT2	Yellow	Negative	Hub gene
	KNL1	Blue	Negative	Correlated genes		PARPBP	Blue	Negative	Hub gene
	CEP55	Green yellow	Negative	Correlated genes		COL5A1	Blue	Negative	Hub gene
	ADIPOQ	Salmon	Positive	Correlated genes		ADAMTS2	Blue	Negative	Hub gene
					BMD pathology	RPS4Y1	Turquoise	Positive	Correlated gene
						KDM5D	Turquoise	Positive	Correlated gene
						CXCL5	Turquoise	Positive	Correlated gene

**Table 2:** GO and KEGG pathway analysis that hub genes were enriched in

Traits	Enrichment	Type	Gene symbol
DMD	Immune response	BP	IRF8,CD33,C3AR1,TLR7,TYROBP,AIF1
	Myeloid leukocyte activation	BP	CD33,C3AR1,TLR7,TYROBP,AIF1
	Regulated exocytosis	BP	CD33,C3AR1,PLEK,TYROBP
	Cell activation	BP	CD33,C3AR1,TLR7,PLEK,TYROBP,AIF1
	Neutrophil degranulation	BP	CD33,C3AR1,TYROBP
	Neutrophil activation involved in immune response	BP	CD33,C3AR1,TYROBP
	Defense response	BP	IRF8,C3AR1,TLR7,TYROBP,AIF1
	Neutrophil activation	BP	CD33,C3AR1,TYROBP
	Neutrophil mediated immunity	BP	CD33,C3AR1,TYROBP
	Granulocyte activation	BP	CD33,C3AR1,TYROBP
	Secretory vesicle	CC	CD33,C3AR1,TYROBP
	Secretory granule	CC	CD33,C3AR1,TYROBP
	Lysosome	CC	C3AR1,TLR7
	Lytic vacuole	CC	C3AR1,TLR7
	Vacuolar part	CC	C3AR1,TLR7
	Cytoplasmic vesicle part	CC	CD33,C3AR1,TLR7,TYROBP
	Vacuole	CC	C3AR1,TLR7
	Ruffle	CC	PLEK;AIF1
	Whole membrane	CC	CD33,C3AR1,TLR7
	Protein-containing complex binding	MF	AIF1
	Lipid binding	MF	PLEK
	*Staphylococcus aureus* infection	KEGG	C3AR1
	Pertussis	KEGG	IRF8
BMD	Collagen fibril organization	BP	COL5A1,LOX,ADAMTS2
	Extracellular matrix organization	BP	CTSK,COL5A1,ELN,LOX
	Positive regulation of phosphatase activity	BP	PLEK
	Positive regulation of integrin activation	BP	PLEK
	Extracellular structure organization	BP	CTSK,COL5A1,ELN,LOX,ADAMTS2
	Cell adhesion	BP	THBS2;PLEK,COL5A1
	Biological adhesion	BP	THBS2,PLEK,COL5A1
	Extracellular matrix	CC	THBS2,COL5A1,ELN,LOX,ADAMTS2
	Collagen-containing extracellular matrix	CC	THBS2,COL5A1,ELN,ADAMTS2
	Basement membrane	CC	THBS2,COL5A1
	Extracellular matrix component	CC	COL5A1
	Cell projection part	CC	PLEK
	Plasma membrane bounded cell projection part	CC	PLEK
	Extracellular matrix structural constituent	MF	COL5A1,ELN,THBS2
	Structural molecule activity	MF	COL5A1,ELN,THBS2
	Protein kinase C binding	MF	PLEK
	Magnesium ion binding	MF	CDK2
	Fibronectin binding	MF	CTSK
	p53 signaling pathway	KEGG	CDK2,BCL2L1
	Protein digestion and absorption	KEGG	COL5A1,ELN
	Rheumatoid arthritis	KEGG	CTSK
	Amyotrophic lateral sclerosis (ALS)	KEGG	BCL2L1
	PI3K-Akt signaling pathway	KEGG	THBS2,CDK2,BCL2L1
BMD age	Collagen fibril organization	BP	COL5A1,COL14A1,ADAMTS2
	Endodermal cell differentiation	BP	COL5A1
	Extracellular matrix organization	BP	COL5A1,COL14A1,ADAMTS2
	Endoderm formation	BP	COL5A1
	Extracellular structure organization	BP	COL5A1,COL14A1,ADAMTS2
	Endoderm development	BP	COL5A1
	Extracellular matrix	CC	COL5A1,COL14A1,ADAMTS2
	Collagen-containing extracellular matrix	CC	COL5A1,COL14A1,ADAMTS2
	Extracellular matrix component	CC	COL5A1,COL14A1
	Extracellular matrix structural constituent	MF	COL5A1,COL14A1
	DNA binding, bending	MF	COL5A1,COL14A1
	Structural molecule activity	MF	COL5A1,COL14A1
	Extracellular matrix structural constituent conferring tensile strength	MF	COL5A1,COL14A1
	Protein digestion and absorption	KEGG	COL5A1,COL14A1

## Discussion

The process of losing muscle function in DMD and BMD patients starts from a very early age and is irreversible, therefore, the genetic intervention for DMD and BMD should be as early as possible, since genetic therapy is unable to restore muscle tissue that has already lost function. The current study employed WGCNA to identify highly correlated hub genes in samples of vastus lateralis from patients with DMD and BMD, and healthy control. As a result, 10 hub genes were summarized for DMD and BMD respectively, 5 hub genes were summarized for BMD age, 5 and 3 highly correlated clustered genes were summarized for in DMD age and BMD pathology, respectively. In addition, 20 GO enrichment terms were found to be involved in DMD, 3 GO enrichment terms were found to be involved in BMD, 3 GO enrichment terms were found to be involved in BMD age.

Ten hub genes were identified for DMD and they are all positively correlated with DMD, among them, 5 were from turquoise module, 3 were from yellow module, 1 was from brown module and the last was from tan module. The five genes from turquoise module were C3AR1(encodes complement component 3a receptor 1), TLR7(encodes toll like receptor 7), IRF8(encodes interferon regulatory factor 8), FYB (encodes FYN binding protein) and CD33(encodes CD33 molecule). C3 molecule is a biomarker for muscle fiber diseases [13, 14]. In addition, it has been demonstrated that C3 gene knockout can relieve muscle pathology in dysferlin-deficient mice [15]. Moreover, scientists have found that histone deacetylase inhibitors, which can attenuate DMD pathology, lowers C3 molecule level in DMD mice [16]. These may suggest the importance of C3AR1 protein and its gene upregulation in DMD. It has been observed that the expression of TLR7 gene increases in DMD mice, the upregulating TLR7 gene expression can induce inflammatory signaling pathway. Moreover, treating DMD mice with TLR7 molecule antagonist can clearly relieve skeletal muscle inflammation and improve muscle force [17]. This indicated the role of TLR7 gene as a potential therapeutic target for DMD. IRF8 protein is a crucial modulator of inflammation in immune cells [18]. FYN protein is a member of Src family kinase, it is also involved in inflammatory signaling pathway [19, 20]. CD33 molecule is a myeloid antigen and play an essential role in the inflammation [21, 22]. Genes from turquoise module mainly participate in immune and inflammation, this suggested immune and inflammation play an important role in DMD, which is consistent with previous studies [23, 24]. Three hub genes for DMD were from yellow module, included PLEK (encodes pleckstrin), TYROBP (encodes TYRO protein tyrosine kinase binding protein) and AIF1(encodes allograft inflammatory factor 1). Pleckstrin is thought to be involved in actin rearrangement [25], in addition, it is associated with platelets adhesion to collagen [26]. TYROBP protein is a part of inflammation signaling pathway that is associated with actin cytoskeleton reorganization [27]. AIF-1 protein is an actin binding protein and may related to actin rearrangement [28, 29]. The hub genes from yellow module are all associated with actin reorganization, this suggested that actin reorganization may play a vital role in DMD, this result is similar to a previous study [30]. LAPTM5(encodes lysosomal protein transmembrane 5) was from brown module, NT5E(encodes 5′-nucleotidase ecto) was from tan module, and they were both hub genes for DMD. Scientists have demonstrated that LAPTM5 gene is closely related to programmed cell death [31]. NT5E gene is mainly expressed in smooth muscle cells [32], its encoding protein can convert adenosine 5′-monophosphate to adenosine and is associated with arterial calcification [33]. It has been observed that arterial stiffness increases in DMD patients [34].

Five genes correlated with DMD age were identified, two of them were from black module, others were from blue, salmon and greenyellow module, respectively. KRT31(encodes keratin 31), KRT33A(encodes keratin 33A) were two correlated genes from black module, ADIPOQ (encodes adiponectin) was from salmon module, the three genes were positively correlated with DMD age. KNL1(encodes kinetochore scaffold 1) and CEP55(encodes centrosomal protein 55) were two genes that negatively correlated with DMD age. It is reported that adiponectin regulates senescence in keratinocytes [35, 36]. Therefore, KRT31, KRT33A and ADIPOQ genes may interact with each other in DMD patients with different age. KNL1 and CEP55 are two genes associated with cellular cycling, and therefore, they may correlate with DMD age. In addition, it is reported that the expression of some centrosomal proteins decreases in muscular dystrophy [37, 38].

COL5A1(encodes collagen type V alpha 1 chain) and ADAMTS2(encodes ADAM metallopeptidase with thrombospondin type 1 motif 2) were common hub genes for BMD and BMD age. The accumulation of collagens is the feature of skeletal muscle fibrosis in BMD patients [39], therefore, collagen associated genes are hub genes and positively correlated with BMD. In addition, it seems that the expression level of collagen including COL5A1 and COL14A1 genes is negatively related to BMD age, a previous study suggested that the collagen level should increase in dystrophic mice [16]. However, we speculated that the possible reason for negatively relationship between collagen gene and BMD age may result from the relatively light symptom in the aging patients with BMD, and the muscle damage is also light, thus causing low collagen expression level compared with younger and severe patients. ADAMTS2 gene is involved in collagen processing [40]. In addition, it is reported that ADAMTS2 gene is also involved in aging [41].

Eight hub genes for BMD were from blue module, and all of them were positively correlated with BMD, these genes included LOX (encodes lysyl oxidase), ELN (encodes elastin), PLEK, IKZF1(encodes IKAROS family zinc finger 1), CTSK (encodes cathepsin K), THBS2(encodes thrombospondin 2), ADAMTS2 and COL5A1. LOX gene is involved in fibrogenesis, as well as collagen and elastin cross-linking, it is observed that its expression level is increased in mice and dogs with muscular dystrophy [42, 43], therefore, LOX, as well as ELN gene may be positively correlated with patients with BMD. It has been observed that overexpression of IKZF1 gene can upregulate matrix metalloproteinase, which plays an important role in BMD [44]. Collagens are major constituents of the extracellular matrix (ECM), while Cathepsin K plays an important role in ECM degradation [45]. Thrombospondin 2 can regulate the production of ECM and LOX protein levels [46]. PARPBP (encodes PARP1 binding protein) is another hub gene that from blue module, it is negatively related to BMD age. It is also associated with ECM [47]. Studies have demonstrated its role in BMD [48, 49]. The genes in blue modules are all involved in ECM, which suggests the vital role of ECM in BMD and BMD age, this is consistent with previous studies [50, 51].

BCL2L1(encodes BCL2 like 1) and CDK2(encodes cyclin dependent kinase 2) were two hub genes from turquoise module and were both negatively correlated with BMD. It has been observed that BCL2L1 protein is a part of signaling pathway that can promote cell division [52]. CDK2 protein participates in cell cycling, the negatively relationship between the two genes and BMD indicates a muscular damage and dystrophy [53]. RPS4Y1(encodes ribosomal protein S4, Y-linked 1), KDM5D(encodes lysine demethylase 5D) and CXCL5(encodes C-X-C motif chemokine ligand 5) were also from turquoise module and all positively correlated with BMD pathology. RPS4Y1 and KDM5D genes are both from Y chromosome. It has been demonstrated that they both participate in cell cycle, which suggests that turquoise module mainly involves in cell cycle [54–56]. ECT2(encodes epithelial cell transforming 2) was from yellow module, it was a hub gene and negatively correlated with BMD age. It is reported that ECT2 gene is an oncogene and associated with senescence [57].

The GO and KEGG pathway analysis of all hub genes for DMD, BMD and age indicated that the enrichment mainly involves immune and inflammation for DMD, while hub genes for BMD mainly enriched in ECM, this is consistent with our analysis of hub gene. This indicated that DMD and BMD may differ in the pathological mechanism, the different pathological mechanisms between the two diseases may provide new pharmaceutical therapy for DMD and BMD. Compared with the previous bioinformatic study using the same dataset [12], only a few hub genes were the same between the two papers, this may lie in the application of whole gene array in their study and DEGs in our study. However, both studies have found the immune system may be involved in DMD, this suggests the its potential key role in DMD.

There still exist several limitations that may influence our results. Firstly, the number of genes in each clustered module was small, and the number of genes in the non-clustered grey module was large, which forced us to analyse all correlated clustered genes instead of only one module. Secondly, the number of correlated clustered genes in DMD age and BMD pathology was too small to conduct enrichment and PPI analysis, in addition, all correlated genes for DMD pathology were from grey module. Thirdly, basic demographics characteristics (such as gender and age) of healthy individuals were not applicable, and the number of the three groups was also not big enough. Lastly, the difference between the selected threshold and the real line in Fig. 3 was larger than was ideal.

## Conclusion

In conclusion, several hub genes are identified for DMD: C3AR1, TLR7, IRF8, FYB and CD33(immune and inflammation associated genes), TYROBP, PLEK, AIF1(actin reorganization associated genes), LAPTM5 and NT5E(cell death and arterial calcification associated genes, respectively). In BMD, a number of hub genes are identified: LOX, ELN, PLEK, IKZF1, CTSK, THBS2, ADAMTS2, COL5A1(ECM associated genes), BCL2L1 and CDK2(cell cycle associated genes). Keratin may play an important role in DMD age, while ECM may play a key role in BMD age, and cell cycle may be associated with BMD pathology. It is important to diagnose and treat DMD and BMD at an early age via the expression level of hub genes. Further studies are required to explore the relevant genes in DMD and BMD, as well as pharmaceutical therapies aimed at these targets.

## Data Availability

As a bioinformatics analysis, there are no patient data sets.

## Abbreviations

BMD: Becker muscular dystrophy
DGC: Dystrophin-glycoprotein complex
DMD: Duchenne muscular dystrophy
FC: Fold change
FDR: False discovery rate
GEO: Gene Expression Omnibus
GO: Gene Ontology
KEGG: Kyoto Encyclopaedia of Genes and Genomes
PPI: Protein–protein interaction
STRING: Search Tool for the Retrieval of Interacting Genes
TOM: Topological overlap matrix
WGCNA: Weighted correlation network analysis
